# miRNA Regulation Network Analysis in Qianliening Capsule Treatment of Benign Prostatic Hyperplasia

**DOI:** 10.1155/2015/365484

**Published:** 2015-07-30

**Authors:** Liya Liu, Yun Wan, Aling Shen, Jinyan Zhao, Jiumao Lin, Xiaoyong Zhong, Yuchen Zhang, Zhenfeng Hong

**Affiliations:** ^1^Academy of Integrative Medicine Biomedical Research Center, Fujian University of Traditional Chinese Medicine, Fuzhou, Fujian 350122, China; ^2^Fujian Key Laboratory of Integrative Medicine on Geriatrics, Fujian University of Traditional Chinese Medicine, Fuzhou, Fujian 350122, China

## Abstract

*Objective*. The objective of this study was to evaluate the molecular mechanism by which Qianliening capsule (QC) treats benign prostatic hyperplasia (BPH). *Methods*. Benign prostatic hyperplasia epithelial cell line BPH-1 was treated with 0, 1.25, 2.5, and 5 mg/mL QC for 48 h, respectively. Evaluation of cell viability and observation of morphologic changes of BPH-1 cell gene expression and miRNA expression profiles were analyzed. Real-time quantitative PCR was used to confirm changes in miRNA and gene expression. GO and KEGG pathway-based approaches were used to investigate biological functions and signaling pathways affected by differentially expressed mRNAs. *Results*. QC inhibited BPH-1 cell proliferation. Differential expression of 19 upregulated and 2 downregulated miRNAs was observed in QC-treated BPH-1 cells compared to untreated control cells. 107 upregulated and 71 downregulated genes were identified between the two groups. Significantly enriched signaling pathways based on deregulated mRNAs were mainly involved in regulation of cell proliferation, apoptosis, and so on. Additionally, miRNA-mRNA network analysis integrated these miRNAs and genes by outlining interactions of miRNA and related genes. *Conclusion*. The study was the first report of differentially expressed miRNA and mRNA in QC-treated BPH-1 cells.

## 1. Introduction

Benign prostatic hyperplasia (BPH) is a pathological overgrowth of the human prostate, a condition that affects a majority of men older than 50 years [1]. Most patients with BPH experience increased resistance in urinary flow leading to urinary tract symptoms (LUTS) including urinary hesitancy, frequent urination, urgency, thin urine flow, and bladder stones [2]. These symptoms greatly affect the physical and mental health of patients as well as their quality of life. Unfortunately, the cellular and molecular mechanisms contributing to BPH development and progression remain unclear.

Microribonucleic acid (miRNA), a 19–24 noncoding family of nucleotide (nt), is processed from 70 to 100 nt double-stranded hairpin precursors by RNase III dicer and endogenously expressed in the RNA-induced silencing complex within the cytoplasm [3]. miRNA recognizes the 3′-untranslated region of target mRNA(s) with imperfect complementarity which causes translational repression or mRNA cleavage [4]. Currently, growing evidence highlights the importance of miRNAs in association with pathophysiologic processes including abnormal cell proliferation and differentiation, invasion, metastasis, and the poor prognosis of various cancers and relative diseases [5–9]. Therefore, it is plausible that certain miRNAs participate in the pathogenesis of BPH, which constitutes the rationale for our research.

The mainstay of pharmacotherapy for BPH is a combination treatment of 5*α*-reductase inhibitors that regulate levels of 5-dihydrotestosterone (DHT) and *α*-adrenergic blockers that inhibit *α*-adrenergic receptors. However, these medications may induce undesired side effects such as orthostatic hypotension, decreased libido and ejaculation, or erectile dysfunction [10–14]. Qianliening capsule (QC) is a traditional Chinese medicine formulation consisting of wine rhubarb, leech, Milkvetch root,* Achyranthes aspera*, and dodders. These components together confer QC properties of heat-clearing, detoxification, promotion of blood circulation, removal of blood stasis, tonifying the kidney, and nourishing vitality (replenishing the kidney qi in Chinese) [15, 16]. Previous studies have demonstrated that QC has significant therapeutic effects on BPH [16–18]. In clinical trials, QC clearly improved a series of lower urinary tract symptoms (LUTS) in BPH patients, such as frequency of urination, urinary urgency, thin urine flow, incontinence, and other voiding disorders [19]. Our preliminary study in a rat model of BPH showed that QC significantly decreased prostatic volume and weight, inhibited prostatic hyperplasia, attenuated abnormal serum levels of estrogen and androgen, regulated the expression of estrogen receptor (ER), androgen receptor (AR), and related mRNA, inhibited the EGF/STAT3 pathway, and reduced expression of proproliferative PCNA, cyclin D1, and CDK4 proteins [15–19]. Moreover, QC effectively inhibited proliferation and promoted apoptosis in human benign prostatic hyperplasia epithelial cells and prostate cells [20, 21]. To more fully clarify the mechanistic effects of QC therapy in the treatment of BPH, we performed the present study to examine the effects of QC on expression of specifically expressed miRNAs, genes, and relevant signaling pathways in our rat model of BPH.

## 2. Materials and Methods

### 2.1. Materials and Reagents

Fetal bovine serum (FBS), Roswell Park Memorial Institute 1640 (RPMI 1640) medium, penicillin-streptomycin, and trypsin-EDTA were purchased from Life Technologies (Carlsbad, CA, USA). A cell proliferation WST-1 assay kit was purchased from Roche Applied Science Gmbh (Mannheim, Germany). TRIzol reagent was purchased from Life Technologies (Carlsbad, CA, USA). Superscript II reverse transcriptase was obtained from the Promega Corporation (Madison, WI, USA). All other chemicals, unless otherwise stated, were obtained from SigmaAldrich (St. Louis, MO, USA).

### 2.2. Preparation of QC

Qianliening capsule (QC, Fujian, China, FDA approval number: Z09104065) was provided by the Academy of Pharmacology of Fujian Chinese Medical University. The drug powder inside the capsule was dissolved in distilled water and stored at −20°C. Working concentrations of QC were prepared by diluting the stock solution in culture medium.

### 2.3. Cell Culture

Benign prostatic hyperplasia epithelial (BPH-1) cells were obtained from Xiangya Cell Center, University of Zhongnan (Hunan, China), and grown in RPMI 1640 medium. RPMI 1640 was supplemented with 10% (v/v) FBS and 100 units/mL penicillin and 100 *μ*g/mL streptomycin. All cells were cultured at 37°C and 5% CO_2_ under humidified environment.

### 2.4. Evaluation of Cell Viability by WST-1 Assay

The influence of increasing QC concentration on the viability of BPH-1 cells was determined using a cell proliferation reagent WST-1 kit. Briefly, BPH-1 cells were harvested from exponential phase cultures growing in RPMI 1640 with 10% FBS, counted, plated in 96-well flat-bottomed microtiter plates (100 *μ*L cell suspensions, 2.5 × 10^4^ cells/mL), and treated with medium containing various concentrations (0 mg/mL, 1.25 mg/mL, 2.5 mg/mL, and 5 mg/mL) of QC. After 48 h, 10 *μ*L WST-1 added to each well and the reaction mixture was incubated at 37°C in a 5% CO_2_ atmosphere for 0.5 h. Sample absorption was measured under a wavelength of 450 nm using a spectrophotometer (Bio Tek Model ELX800, USA), and the results were compared as percentages of control cells.

### 2.5. Observation of Morphologic Changes

BPH-1 cells were seeded into 6-well plates at a density of 1.0 × 10^5^ cells/well in 2 mL medium. The cells were treated with various concentration (0, 1.25, 2.5, 5 mg/mL) QC for 48 h. Cell morphology was observed using a phase-contrast microscope (Leica, Mannheim, Germany). All images were acquired at 400x magnification.

### 2.6. RNA Extraction for Microarray and Real-Time PCR Analysis

BPH-1 cells were seeded into 75 cm^2^ flasks at a density of 5 × 10^5^ cells/flask in 15 mL medium. The cells were treated with 2.5 mg/mL QC for 48 h and total RNA, including small RNA, was isolated with TRIzol reagent. RNA purity and concentration were determined via OD 260/280 readings using a spectrophotometer (NanoDrop 2000c). Concerning differential miRNAs and mRNAs with comparative microarray-determined expression levels over 2.0 or below 0.5, real-time PCR assays were utilized to detect and quantify the differential pre-miRNAs and mRNAs as previously described [22, 23] using SYBR Green dye. U6 was used as an internal control. The threshold cycle (CT) is defined as the fractional cycle number at which fluorescence passes a fixed threshold. The miRNA and mRNA expression levels were normalized to U6, relative expression was calculated using the comparative ΔΔCT method, and values were expressed as 2^−ΔΔCT^ [24].

### 2.7. miRNA Expression Microarray Analysis

A GeneChip miRNA 3.0 array (Affymetrix, Santa Clara, CA) was hybridized using 500 ng total RNA per standard Affymetrix protocols. The same RNA preparations used in the mRNA microarray analysis were used for miRNA array analysis. Data extraction was completed using Affymetrix Command Console software. Raw data was analyzed by the following workflow: background detection, RMA global background correlation, quantile normalization, median polish, and log⁡2 transformation with miRNA QC tool software.

### 2.8. Gene Expression Microarray Analysis

Microarray analysis was accomplished by hybridization to a GeneChip PrimeView human gene expression array per manufacturer's instructions (Affymetrix, Santa Clara, CA). Genes that underwent at least a 2-fold change between treatment groups were selected for real-time PCR validation.

### 2.9. Gene Ontology and Kyoto Encyclopedia of Genes and Genomes Pathway Analysis Based on Differentially Expressed mRNAs

Gene ontology (GO) (http://www.geneontology.org/) and Kyoto Encyclopedia of Genes and Genomes (KEGG) databases (http://www.genome.ad.jp/kegg/) were used to investigate biological functions and signaling pathways affected by differentially expressed mRNAs [25–27]. A *P* value less than 0.05 was considered statistically significant. Meanwhile, we constructed the miRNA-mRNA regulatory network basis on different miRNAs and their targets; the miRNA-mRNA interaction network, representing critical miRNAs and their targets, was established according to miRNA degree [28]. Networks of miRNA and genes from differentially expressed miRNA and mRNA in the two groups were visualized with the Cytoscape tool [28].

### 2.10. Statistical Analysis

Data are presented as means ± SD for the indicated number of independently performed experiments and analyzed using the SPSS software package for Windows (version 16.0). Statistical analysis of the data was performed with Student's *t*-test and ANOVA. Differences with *P* < 0.05 were considered to be statistically significant.

## 3. Results

### 3.1. QC Inhibits Proliferation of BPH-1 Cells

**Figure 1:**
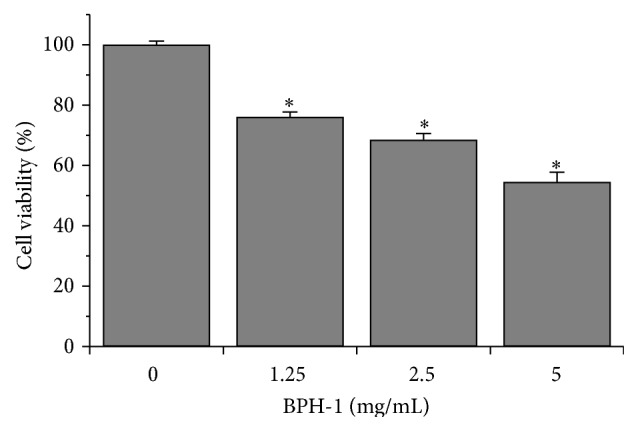
Effect of QC on BPH-1 cell viability. Cell viability was determined by the WST-1 assay after BPH-1 cells were treated with various concentrations of QC for 48 h. The data were normalized to the viability of control cells (100%). Data are the averages with SD (error bars) from 3 independent experiments. ^*∗*^ *P* < 0.05, versus control cells.

**Figure 2:**
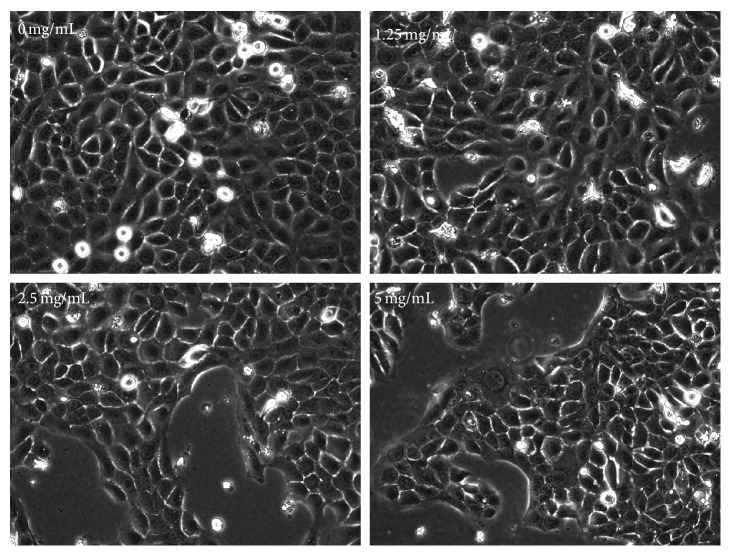
QC treatment and BPH-1 cell confluence. QC treatment dose-dependently decreased the cell density of BPH-1 cells, which was observed via phase-contrast microscopy. Images are representatives of 3 independent experiments.

The effect of QC on BPH-1 cell viability was determined by the WST-1 assay. As shown in Figure 1, treatment with 1.25–5 mg/mL QC for 48 h reduced cell viability by 50%–80% in a dose-dependent manner compared with untreated control cells (*P* < 0.05). To further verify these results, we evaluated the effect of QC on BPH-1 cells confluence via phase-contrast microscopy. QC treatment gradually decreased monolayer confluence with increasing drug concentration (Figure 2). Taken together, these data demonstrate that QC inhibited BPH-1 cell proliferation.

### 3.2. miRNA Expression Profiling

**Figure 3:**
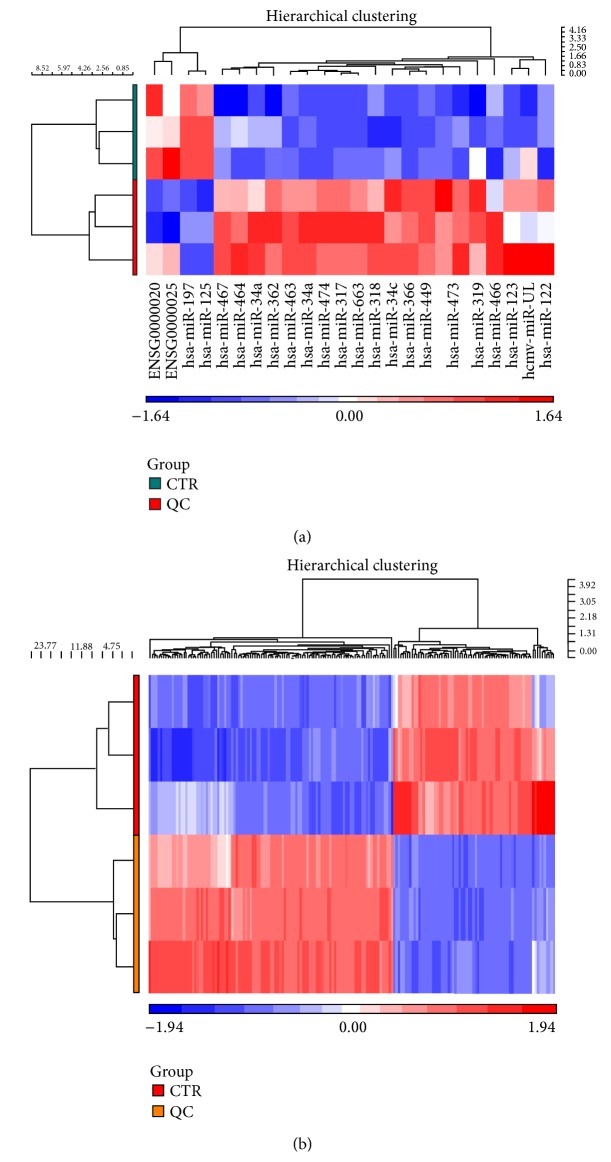
Cluster analysis of differentially expressed miRNA and mRNA with and without QC treatment. (a) miRNA signature in BPH-1 cells treated with QC and controls. (b) mRNA signature in BPH-1 cells treated with QC and controls. Signal intensity was expressed as a log⁡2 ratio between QC treatment and controls. Bright blue, underexpression; white, no change; bright red, overexpression. *P* < 0.05.

The GeneChip miRNA array version 3.0 (Affymetrix) was used to profile miRNAs that were differentially expressed between BPH-1 cells treated with QC (2.5 mg/mL) and control cells. All of the 1733 miRNAs that were probed, 21 were differentially expressed in QC-treated BPH-1 cells (Table 1). These are shown visually in a cluster analysis (Figure 3(a)). 19 of 21 were overexpressed whereas 2 of 21 were underexpressed. The miR-34 family (including miR-34a and miR-34c), miR-3185, miR-663, and miR-125b, are involved in apoptosis and proliferation [29–34]; miR-122 and miR-1231 are associated with liver disease [35–37], and miR-3663 is associated with skin or smooth muscle cancer [38, 39]. However, the biological functions of the other miRNAs that significantly changed in the miRNA array have not been reported previously and require further study.

### 3.3. Gene Expression Profiling

To gain insight into the mechanisms of QC action, we conducted gene expression profiling by GeneChip PrimeView human gene expression array, as shown visually via cluster analysis (Figure 3(b)). Significant changes in 178 genes were observed between QC-treated BPH-1 cells and controls, of which 107 genes were upregulated and 71 genes were downregulated (Table 2). Genes that were identified as differentially expressed exhibited >2.0-fold change between the BPH-1 cells treated with QC and control cells.

### 3.4. Validation of Differentially Expressed miRNAs and Genes

**Figure 4:**
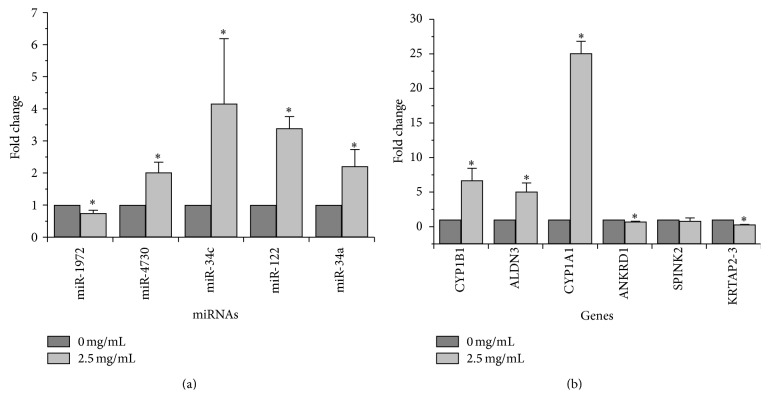
Confirmation of miRNA and mRNA expression by real-time PCR. (a) The miRNA levels of different miRNA in QC-treated and untreated cells were determined by real-time PCR. (b) The mRNA levels of different mRNA in QC-treated and untreated cells were determined by real-time PCR. U6 was used as the internal controls for the real-time PCR, respectively. Images are representative from 3 independent cell-based experiments.

Real-time PCR was used to validate the microarray analysis findings. The relative expressions, expressed as percent change from controls of the five chosen miRNAs (miR-34a, miR-122, miR-34c, miR-4703, and miR-1972), are shown in Figure 4(a). These data confirmed that miR-34a, miR-122, miR-34c, and miR-4703 were increased in QC-treated BPH-1 cells whereas miR-1972 expression was decreased (*P* < 0.05 for all). Meanwhile, we randomly selected 8 different genes (CYP1B1, ALDN3, CYP1A1, ANKRD1, KRTAP2-3, and SPINK2), and these data confirmed that CYP1B1, ALDN3, CYP1A1, and ANKRD1 were overexpressed in two groups whereas KRTAP2-3 expression was decreased (*P* < 0.05 for all) (Figure 4(b)). SPINK2 expression, which was not significantly different from controls, was also confirmed not to be significantly different between the two groups.

### 3.5. GO and KEGG Pathway Analysis of Deregulated mRNAs

**Figure 5:**
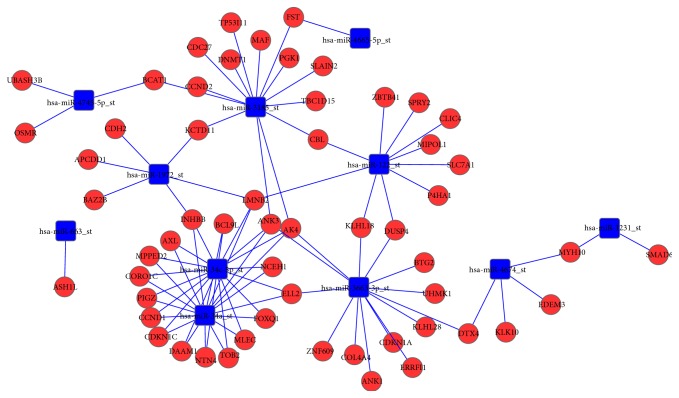
Interaction network of upregulated genes and downregulated miRNAs/mRNAs in BPH-1 cells treated with QC (2.5 mg/mL) and control cells. Blue box nodes represent miRNA, and red cycle nodes represent mRNA. Edges show the inhibitory effect of miRNA on mRNA. The miRNA-mRNA network was visualized by Cytoscape tool.

GO analysis showed that the differentially expressed mRNAs between QC-treated BPH-1 cells and the control group were significantly enriched in oxidation reduction, regulation of cell proliferation, immune response, regulation of apoptosis, regulation of cell death, and so forth. KEGG pathway analysis indicated that the deregulated mRNAs between the two cell groups were mainly involved in metabolism of xenobiotics by cytochrome P450, steroid hormone biosynthesis, drug metabolism, and TGF-beta signaling pathway (*P* value <0.05 after multiple testing corrections) (Tables 3-4). Additionally, miRNA-mRNA network analysis integrated these miRNAs and genes by outlining the interactions between them (Figure 5).

## 4. Discussion

Qianliening capsule (QC) is a traditional Chinese formulation that has long been used to clinically treat benign prostate hyperplasia (BPH). However, BPH is a complex disease as its pathogenesis and progression are associated with multiple factors, genes, and signal transduction pathways, all of which are further highly regulated by an miRNA regulatory network. As such, much effort has been placed on understanding the mechanisms underlying the disease process and treatment mechanisms of QC in BPH. Research on miRNAs in various diseases is still in its infancy, although exciting findings including the link between miRNAs and prostate cancer have recently been reported [40]. To our knowledge, however, there have been no reports on the association between miRNAs and any spectrum of BPH. In order to identify potentially unique miRNA expression profiles, we jointly employed miRNA and gene microarray analysis to detect aberrantly expressed miRNA and mRNA in BPH-1 cells.

Hundreds of miRNAs have been shown to play important roles in regulating gene expression through degradation of mRNA or repression of translation in a variety of model systems [41, 42]. Microarray gene expression profiling has improved our understanding of BPH biology and allowed the development of multigene “signatures” to predict outcome and response to systemic therapies [32]. Through comprehensive array profiling and analysis of miRNA and gene expression levels, we have identified putative candidate genes and pathways that mediate QCs effects in BPH. Genes differentially expressed in response to QC treatment included those involved in cell growth, proliferation, and apoptosis, common characteristics in hyperplasic diseases.

Our preliminary research showed that QC could inhibit proliferation and promoted apoptosis* in vivo* and* in vitro* [20, 21]. According to our miRNA array results, most of the miRNAs have previously been shown to be associated with apoptosis, proliferation, and metabolism. For example, the miR-34 family, including miR-34a and c, has been known to regulate several cellular events, including the cell cycle, cell migration, and apoptosis [29, 30]. miR-663 is involved in the TGF-*β* signaling pathway [32, 33]. miR-3185 likely plays an important role in regulating MAPK signaling [31]. miR-122 is suggested to regulate many target genes in lipid and cholesterol metabolism [35, 36]. Interestingly, among 242 altered genes identified by our gene microarray experiment, more than 30 genes were involved in apoptosis and proliferation. Their cellular functions encompass regulation of cell proliferation, cell cycle, and cell death. More than 10 genes were involved in metabolism (metabolism of xenobiotics and drug metabolism). The miRNA-mRNA interaction network analysis further integrated bioinformatic findings and then outlined the primary different miRNAs and their major target gene(s). It is of great interest to determine in future studies whether these same sets of miRNAs and their target genes could be biomarkers that are associated with mechanisms by which QC treats BPH.

## Figures and Tables

**Table 1:** Differentially expressed miRNA between the BPH-1 cells treated with QC (2.5 mg/mL) group and control group.

miRNA	Fold change (QC versus CTR)
miR-3663-3p	5.25562
miR-4730	5.15551
miR-34c-5p	3.4525
miR-122	3.40821
miR-4674	3.30796
miR-34a	3.28902
miR-4734	3.19241
miR-3178	3.1713
miR-4497	3.164
miR-4649-5p	2.96402
miR-3185	2.96402
miR-3621	2.90446
miR-663	2.60666
miR-4634	2.55855
miR-4745-5p	2.31046
miR-34a	2.21767
miR-4665-5p	2.11388
miR-3195	2.06861
miR-1231	2.04573
miR-125b-1	−2.47347
miR-1972	−2.76246

Positive value indicated upregulation and negative value indicated downregulation. miRNA with expression fold change >2 and *P* value < 0.05 was considered statistically significant. QC: BPH-1 cells treated with QC (2.5 mg/mL); CTR: control group.

**Table 2:** Differentially expressed genes between the BPH-1 cells treated with QC (2.5 mg/mL) group and control group.

Genes	Fold change (QC versus CTR)
CYP1A1	12.8207
CYP1B1	8.49316
ALDH3A1	8.25975
AKR1C1	6.11892
AKR1C3	4.37771
EDIL3	3.87485
SCGB1A1	3.78913
SEMA5A	3.39468
DOCK8	3.31441
MPPED2	3.14276
SPINK6	−5.87906
KRTAP2-3	−4.78595
ANKRD1	−4.25824
S1PR1	−3.49797
SPINK7	−3.34239
DUSP6	−3.24443
CA9	−3.20190
PTGS2	−3.06597
BNIP3	−2.82441
IL7R	−2.81757

Positive value indicated upregulation and negative value indicated downregulation. Genes expression fold change >2 and *P* value < 0.05 were considered statistically significant. QC: BPH-1 cells treated with QC (2.5 mg/mL) group; CTR: control group. The top 10 different genes are shown.

**Table 3:** List of enriched KEGG pathways of differentially expressed mRNAs in BPH-1 cells treated with QC (2.5 mg/mL) and control cells.

Pathway ID	Definition	Gene count	*P *value
hsa00980	Metabolism of xenobiotics by cytochrome P450	10	1.87*E* − 09
hsa00140	Steroid hormone biosynthesis	6	5.04*E* − 05
has00982	Drug metabolism	5	0.00223
hsa04350	TGF-beta signaling pathway	4	0.04332

Enriched KEGG pathways were used for analysis of the differentially expressed mRNAs between two groups; *P* values after multiple testing corrections <0.05.

**Table 4:** List of enriched GOs of differentially expressed mRNAs in BPH-1 cells treated with QC (2.5 mg/mL) and control cells.

ID	Definition	Gene count	*P* value
GO:0055114	Oxidation reduction	18	1.20*E* − 06
GO:0042127	Regulation of cell proliferation	15	8.27*E* − 04
GO:0006955	Immune response	14	7.61*E* − 04
GO:0042981	Regulation of apoptosis	14	0.002994
GO:0043067	Regulation of programmed cell death	14	0.00326
GO:0010941	Regulation of cell death	14	0.003365
GO:0008219	Cell death	12	0.009416
GO:0016265	Death	12	0.009889
GO:0012501	Programmed cell death	11	0.00846
GO:0010033	Response to organic substance	11	0.024364
GO:0043065	Positive regulation of apoptosis	10	0.002571
GO:0043068	Positive regulation of programmed cell death	10	0.002694
GO:0010942	Positive regulation of cell death	10	0.002778
GO:0009611	Response to wounding	10	0.009872
GO:0006915	Apoptosis	10	0.021064
GO:0030182	Neuron differentiation	9	0.009842
GO:0042493	Response to drug	8	6.67*E* − 04
GO:0044092	Negative regulation of molecular function	8	0.007664
GO:0022403	Cell cycle phase	8	0.022599
GO:0008284	Positive regulation of cell proliferation	8	0.022599

Enriched GOs were used for analysis of the differentially expressed mRNAs between two groups; *P* values after multiple testing corrections <0.05.

## Abbreviations

BPH: Benign prostatic hyperplasia
QC: Qianliening capsule
miRNAs: MicroRNAs.
